# Cardiovascular and Cutaneous Responses to the Combination of Alcohol and Soft Drinks: The Way to Orthostatic Intolerance?

**DOI:** 10.3389/fphys.2017.00860

**Published:** 2017-11-10

**Authors:** Claire Maufrais, Nathalie Charriere, Jean-Pierre Montani

**Affiliations:** Division of Physiology, Laboratory of Integrative Cardiovascular and Metabolic Physiology, Department of Medicine, University of Fribourg, Fribourg, Switzerland

**Keywords:** alcohol, sugar, hemodynamics, active standing, cutaneous blood flow

## Abstract

**Aim:** Acute ingestion of alcohol is often accompanied by cardiovascular dysregulation, malaise and even syncope. The full hemodynamic and cutaneous responses to the combination of alcohol and sugar (i.e., alcopops), a common combination in young people, and the mechanisms for the propensity to orthostatic intolerance are not well established. Thus, the purpose of this study was to evaluate the cardiovascular and cutaneous responses to alcopops in young subjects.

**Methods:** Cardiovascular and cutaneous responses were assessed in 24 healthy young subjects (12 men, 12 women) sitting comfortably and during prolonged active standing with a 30-min baseline and 130 min following ingestion of 400 mL of either: water, water + 48 g sugar, water + vodka (1.28 mL.kg^−1^ of body weight, providing 0.4 g alcohol.kg^−1^), water + sugar + vodka, according to a randomized cross-over design.

**Results:** Compared to alcohol alone, vodka + sugar induced a lower breath alcohol concentration (BrAC), blood pressure and total peripheral resistance (*p* < 0.05), a higher cardiac output and heart rate (*p* < 0.05) both in sitting position and during active standing. In sitting position vodka + sugar consumption also led to a greater increase in skin blood flow and hand temperature (*p* < 0.05) and a decrease in baroreflex sensitivity (*p* < 0.05). We observed similar results between men and women both in sitting position and during active standing.

**Conclusion:** Despite lower BrAC, ingestion of alcopops induced acute vasodilation and hypotension in sitting position and an encroach of the hemodynamic reserve during active standing. Even if subjects did not feel any signs of syncope these results could be of clinical importance with higher doses of alcohol or if combined to other hypotensive challenges.

## Introduction

Acute alcohol consumption in social amounts is frequent in young people (Kuntsche et al., 2005). Many studies analyzed the acute cardiovascular response to alcohol at relatively moderate doses (0.3–1.0 g.kg^−1^ body weight) corresponding to social drinking. In healthy normotensive subjects, it is characterized by an increase in heart rate (HR), small early (Iwase et al., 1995), late (Randin et al., 1995) or no changes in blood pressure (BP) (Kupari, 1983; van de Borne et al., 1997; Spaak et al., 2008, 2010; Carter et al., 2011), slightly elevated values of cardiac output (CO) and systemic vasodilation (Kupari, 1983), along with activation of the sympathetic nervous system (Iwase et al., 1995; Randin et al., 1995; van de Borne et al., 1997; Hering et al., 2011) and evidence of some vagal withdrawal (Koskinen et al., 1994; Spaak et al., 2010). Interestingly, alcohol may decrease myocardial contractility in healthy young people (Kelly et al., 1996), already evident at concentrations corresponding to the legal driving limit of 0.5^0^/_00_ common in most Western Europe. In addition, alcohol depresses the vasoconstrictor response to noradrenaline infusion (Eisenhofer et al., 1984) and may interfere with the autonomic nervous system, as it disrupts the vasoconstrictor response to orthostatic stress (Narkiewicz et al., 2000; Carter et al., 2011), and impairs baroreflex function (Abdel-Rahman et al., 1987; Carretta et al., 1988). In this context, it comes to no surprise that alcohol consumption may induce orthostatic hypotension, even in young, healthy subjects. Indeed, healthy young subjects ingesting alcohol (at 1 g.kg^−1^ body weight, in 400 ml water) exhibited a much larger decrease in BP with orthostatic stress, induced by head-up-tilt and graded lower body negative pressures, than when ingesting water (Narkiewicz et al., 2000). Interestingly, the increase in HR was similar in both sessions, despite quite different drops in BP, suggesting that alcohol inhibits the central nervous response to orthostasis.

Although, alcohol could be ingested in various forms (e.g., wine, beer, or hard liquors), alcoholic drinks combine hard liquors (such as vodka) with fruit juice or other types of sugary drinks. Indeed, one way to circumvent legislation of selling hard liquors to underage people or in order to appeal to a younger generation (particularly young women) without the stigma of “hard liquor drinking,” is to propose pre-mixed, ready-to-use drinks combining distilled alcohol with added ingredients such as fruit juice, sugars and flavoring agents. Moreover, in those events, acute alcohol consumption in young people is often taken on a relatively empty stomach increasing the systemic availability of alcohol (Oneta et al., 1998).

Although the effects of acute alcohol consumption have been well studied in healthy individuals, including an orthostatic stress (Narkiewicz et al., 2000; Carter et al., 2011), the interaction of sugary drinks with alcohol on cardiovascular regulation has not been well characterized. In most cardiovascular studies, alcohol is given alone (diluted with water, with some sweetening agent or diluted in some fruit juice). We are not aware of any study that has evaluated the interaction of alcohol and sugar with respect to systemic vasodilatation, autonomic response and, most importantly, in the response to prolonged active standing. Yet, both alcohol (Kupari, 1983) and sugars as glucose (Brown et al., 2008; Grasser et al., 2014) or sucrose (Grasser et al., 2014), when ingested acutely, decrease total peripheral resistance (TPR), partly due to the promotion of insulin secretion (Blaak and Saris, 1996; Steiner et al., 2015), a well-known vasodilator (Taddei et al., 1995; Muniyappa et al., 2007). In this context, the purpose of this study was to evaluate the interaction of alcohol consumption with sugary drinks in healthy young male and female subjects on the cardiovascular system and on the cutaneous response. We hypothesized that the vasodilatory properties of alcohol and the alcohol-induced dysregulation of autonomic tone are potentiated by the concomitant ingestion of sugary drinks in young people (simulating alcopops ingestion), and thus that the combination of sugars with alcohol will (1) accentuate the systemic vasodilation and (2) increase orthostatic intolerance.

## Materials and methods

### Subjects

Twenty-four subjects (12 men and 12 women) of European descent were recruited from our local University student population and their friends. The mean (±standard deviation) age of the participants was 23.3 ± 2.2 years, weight 62.9 ± 10.1 kg and body mass index 21.8 ± 2.1 kg.m^−^2. Exclusion criteria included those with a body mass index greater than 30 kg.m^−2^, competition athletes and individuals with a daily exercise workload exceeding 60 min per day. None of the subjects had any diseases or were taking any medication affecting cardiovascular or autonomic regulation. Between 2 and 5 days before the first test day, the participants visited the laboratory to complete a questionnaire regarding their lifestyle and medical history, and to familiarize themselves with the experimental procedures and equipment. After voiding the bladder, body weight and height were measured using a mechanical column scale with integrated stadiometer (Seca model 709, Hamburg, Germany), body composition using a multi-frequency bioelectrical impedance analysis (Inbody 720, Biospace Co., Ltd., Seoul, Korea), and waist circumference and abdominal fat percentage by bioelectrical impedance analysis using ViScan (Tanita Corporation, Tokyo, Japan), which has been shown to be accurate both for the measurement of waist circumference (Schutz et al., 2012) and for predicting total abdominal fat when validated against Magnetic Resonance Imaging techniques (Browning et al., 2010; Thomas et al., 2010). All participants were requested to avoid alcohol or caffeine for at least 24 h prior to the test. Furthermore, to minimize the effect of physical activity on the morning of each test day, participants were requested to use motorized public transport instead of walking or cycling to reach the laboratory. Written informed consent was obtained from each test subject. The study protocol complied with the Declaration of Helsinki and received local ethics committee approval (Commission cantonale d'éthique de la recherche sur l'être humain, CER-VD 105/15).

### Study design

**Figure 1:**
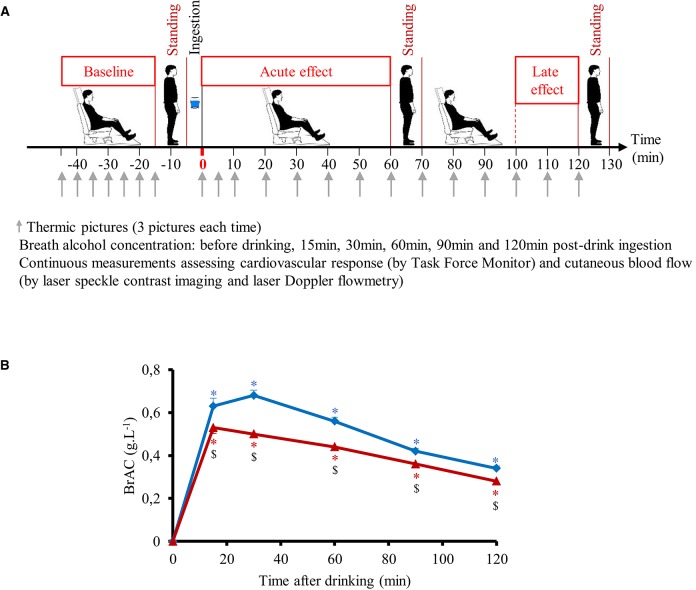
**(A)** Study design including the different periods of sitting and active standing. **(B)** Time course of the changes in breath alcohol concentration (BrAC) after drinking water + vodka (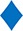) or water + vodka + sugar (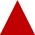). BrAC at 15 min includes 21 subjects because BrAC at this timepoint was not measured in the first three subjects included in the study. All other timepoints include 24 subjects. ^*^p < 0.05 significant differences over time from baseline values; ^$^*p* < 0.05 significant different between responses to the drinks.

All experiments took place in a quiet, temperature-controlled (20–22°C) laboratory and started between 08.00 and 09.00 a.m. On the day of the experiment, after an overnight (12-h) fast the subject took at around 07.00 a.m. a light standardized breakfast provided by us, consisting of one mini-pack of 33 cl of commercial light ice tea (33 kcal, 8 g carbohydrates/6.6 g sugar) and two cereal bars (total of 150 kcal, 39 g carbohydrates/12 g sugar), to avoid that consumption of alcohol in the same morning were done on an empty stomach. Every subject attended four separate experimental sessions (each session separated at least by 2 days) according to a randomized crossover study. Randomization was performed using a random sequence generator (http://www.random.org/sequences/) where the session order was determined for 24 test subjects before the study started. Women were only tested during the follicular phase of their menstrual cycle. The test subjects were not allowed to know the order of their sessions in advance. On arrival at the laboratory, subjects were asked to empty their bladders if necessary and to sit in a comfortable armchair. The cardiovascular monitoring equipment was then connected. Following a variable period for reaching cardiovascular and metabolic stability (usually between 10 and 15 min), and after a stable baseline recording of at least 30 min, the subjects made an orthostatic test consisting of active standing from the sitting position, maintained during 10 min, and then returning to a sitting position (Figure 1A). Then the subjects ingested one of the following four drinks at a temperature of around 10°C (at a convenient pace over 5 min): (1) 390 mL distilled water + 10 mL lemon juice (W), (2) 48 g sucrose + 10 mL lemon juice, diluted in distilled water up to a total volume of 400 mL (S), (3) vodka (40% alcohol per volume, given at 1.28 mL.kg^−1^ of body weight, providing 0.4 g alcohol.kg^−1^) + 10 mL lemon juice, diluted in distilled water up to 400 mL (V), (4) 48 g sucrose + 40% vodka (at 1.28 mL.kg^−1^) + 10 mL lemon juice, diluted in distilled water up to 400 mL (V+S). Hemodynamic monitoring continued for another 130 min post-drink ingestion (Figure 1A) with a 10 min orthostatic test at 60 and 120 min post-drink ingestion. Throughout the procedures, subjects were permitted to watch neutral documentaries on a flat TV screen set at eye level. Breath alcohol concentration (BrAC, with Ethylometer Model 6820, Dräger SA, Germany) was determined before drinking and at 15, 30, 60, 90, and 120 min post-drink. BrAC was determined again just before the subject left the laboratory at the end of the experiment, to document the last BrAC value and to ensure that all subjects were well under the swiss legal limit of 0.5 g alcohol.L^−1^ blood.

### Cardiovascular recordings

Cardiovascular recordings were performed using a Task Force Monitor (CNSystems, Medizintechnik, Graz, Austria) with data sampled at a rate of 1,000 Hz. HR was recorded by a standard 4-lead electrocardiogram. Continuous BP was recorded using the vascular unloading technique from either the index or middle finger (automatically finger switch every 30 min) of the right hand and was automatically calibrated/corrected to oscillometric brachial BP measurements on the left arm. Stroke volume (SV) was assessed on a beat-to-beat basis by impedance cardiography, using three electrodes, placed on the neck and thorax. High frequency (HF: 0.17–0.40 Hz) power components of RR intervals (HF_RRI) were evaluated and given in absolute values (ms2). Keeping in mind the limitations (Parati et al., 2006), we used changes in the HF range of HR variability to assess parasympathetic activity. Baroreflex sensitivity (BRS) was determined from spontaneous fluctuations in BP and cardiac interval using the sequence technique (Bertinieri et al., 1985).

### Cutaneous blood flow and skin temperature

Skin blood flow (SkBf) was recorded non-invasively throughout the whole experiment (except during the orthostatic test) by laser Doppler flowmetry (LDF) (Perimed, Periflux System PF5001, Järfälla, Sweden) and laser speckle contrast imager (LSCI) (PeriCam PSI System, Perimed). The probe of the LDF was set on the dorsum of the left hand between the thumb and the index finger as described previously (Girona et al., 2014), with a within-subject variability for the baseline period between the drinks to be about 23% estimated from our previous study (Girona et al., 2014). However, rather than comparing baseline across separate days, we were interested to monitor overall changes from baseline during the same session, with inherently less variability. LSCI data have shown to have excellent reproducibility (Roustit et al., 2010; Humeau-Heurtier et al., 2014). The laser head of LSCI was placed 35 cm above the skin. On each LSCI recording, a rectangle region of interest—defined as a skin area of interest—was set on the back of the left hand to correspond to a 4 × 4 cm area of skin (i.e., larger than the 10 mm2 recommended, Rousseau et al., 2011).

We made 3 thermographic pictures with FLIR ex (FLIR Systems) of the left hand every 5 min during baseline and the first 10 min post-drink and then every 10 min until the end of the experiment (Figure 1A). Skin temperature was assessed by means of the 3 thermal imaging using a specific software (FLIR Tools version 5.3, FLIR Systems). On each picture, we defined a specific area of interest: 1 cm circle at the top of the third finger and a 5 × 5 cm area on the middle of back of the hand.

### Data analysis

Values of cardiac interval, systolic BP (SBP), diastolic BP (DBP), SV, SkBF, and skin temperatures were averaged every 15 min during the baseline period. Then, these data were averaged from 0–10, 10–20, 20–40, and 40–60 min post-drink period to analyze the acute effects of the drink and 100–120 min post-drink period to assess the late effects of the drink. Cardiac output (CO) was derived as the product of SV and HR, where HR was calculated from the appropriate cardiac interval. TPR was calculated as mean blood pressure (MBP) divided by CO, where MBP was calculated as the result of DBP + 1/3 (SBP-DBP). Double (rate pressure) product (DP) was calculated as HR x SBP and provides valuable information for the oxygen consumption of the myocardium (van Vliet and Montani, 1999).

### Statistical analysis

The number of required subjects was determined by power analysis using the Web software (http://www.statisticalsolutions.net/pssZtest_calc.php), based on a physiologically relevant 5 mmHg change in MAP and a conservative standard deviation of 6 mmHg of the population, based on our previous studies. We chose a type I error (α) of 0.05 and a desired power (1-β) of 0.80, suggesting that a total number of 12 subjects per gender would be required. All values in the text, table and in figures are expressed as mean ± SEM, unless otherwise specified. Statistical analysis was performed using statistical software (Statview version 5.0, SAS Institute Inc., Cary, NC). To test for changes over time from baseline level and to compare mean changes between the drink types, we used two-way ANOVA for repeated measures with time and treatment (drink type) and gender as within-subject factors with *post-hoc* PLSD of Fisher when appropriate. Statistical significance for all analyses was considered at *p* < 0.05.

## Results

### Subject characteristics

The test subject characteristics are presented in Table 1. Baseline pre-drink values on all four test days were similar for hemodynamic measurement parameters, hand and finger temperature, and skin perfusion. No subject reported gastrointestinal symptoms or other unpleasant effects after ingestion of the drinks. After dilution of vodka into water, the concentration of alcohol in the beverage was 10.2 ± 0.4% with higher concentrations for men (11.6 ± 0.6%) than for women (8.7 ± 0.3%, *p* < 0.05).

**Table 1:** Baseline hemodynamic and cutaneous data recorded prior to drink ingestion.

	**Water only**	**Sugar**	**Vodka**	**Vodka + Sugar**
Mean blood pressure (mmHg)	79 ± 2	79 ± 2	81 ± 1	79 ± 2
Cardiac output (L.min^−1^)	5.1 ± 0.1	4.9 ± 0.1	4.9 ± 0.1	5.1 ± 0.1
Total peripheral resistance (mmHg.L^−1^.min)	15.6 ± 0.4	16.3 ± 0.5	16.7 ± 0.4	15.7 ± 0.4
Heart rate (beats.min^−1^)	70 ± 2	70 ± 1	69 ± 2	70 ± 1
Stroke volume (mL)	73 ± 2	71 ± 2	72 ± 2	73 ± 2
Double product (mmHg.beats.min^−1^)	5, 501 ± 178	5, 547 ± 173	5, 608 ± 113	5, 542 ± 166
Contractility index (1,000.s^−1^)	52 ± 3	49 ± 3	50 ± 3	51 ± 2
BRS (ms.mmHg^−1^)	21 ± 2	20 ± 2	19 ± 2	19 ± 2
HF RRI (ln.ms2)	6.0 ± 0.2	5.9 ± 1.2	6.0 ± 0.9	5.9 ± 1.1
Skin blood flow by LSCI (A.U.)	49 ± 4	47 ± 2	48 ± 2	48 ± 2
Skin blood flow by LDF (A.U.)	41 ± 4	45 ± 6	42 ± 4	49 ± 4
Finger temperature (°C)	34.4 ± 0.6	34.5 ± 0.5	34.0 ± 0.9	34.3 ± 0.6
Hand temperature (°C)	35.3 ± 0.3	35.2 ± 0.3	35.0 ± 0.5	35.0 ± 0.3

*BRS, baroreflex sensitivity; HF_RRI, High frequency power components of RR intervals; LDF, laser Doppler flowmetry; LSCI, laser speckle contrast imaging*.

### Breath alcohol concentration

**Figure 2:**
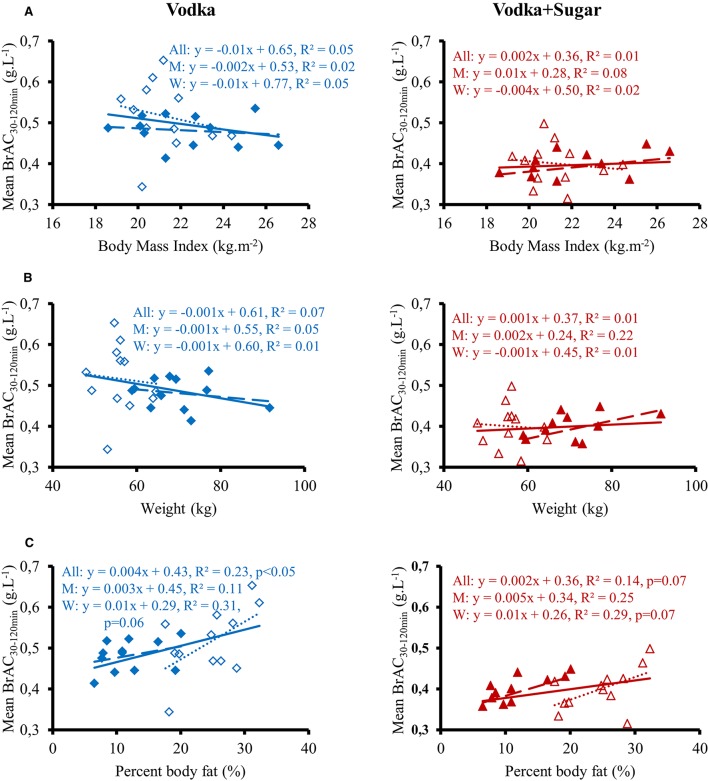
Correlation between mean BrAC_30−120min_ for 12 men (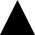, **– –**) and 12 women (Δ, ……) and body mass index **(A)**, weight **(B)**, and percent body fat **(C)** after drinking vodka (left panel) and vodka + sugar (right panel).

Time course of the changes in mean BrAC are presented in Figure 1B. BrAC at each time point and mean BrAC over the test (averaged from 30 to 120 min) were higher after drinking V compared to V+S (0.51 ± 0.01 g.L^−1^ vs. 0.40 ± 0.01 g.L^−1^, respectively, *p* < 0.001). We did not observe any gender difference in BrAC. Correlation between mean BrAC over 30–120 min (BrAC_30−120min_) and anthropometric data are presented in Figure 2. Mean BrAC_30−120min_ tended to be positively correlated to percent body fat in women after drinking V (*R*2 = 0.31, *p* = 0.06) and V+S (*R*2 = 0.29, *p* = 0.07).

### Continuous cardiovascular responses in sitting position

**Figure 3:**
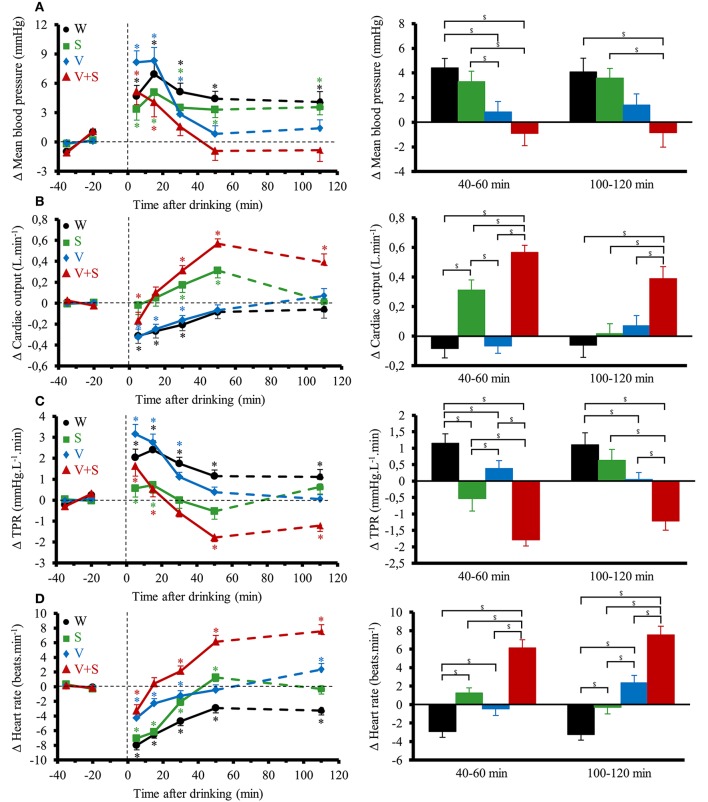
**(A–D)** Left panel: time course of the changes in mean blood pressure **(A)**, cardiac output (CO) **(B)**, total peripheral resistance (TPR) **(C)**, and heart rate **(D)**. Right panel: mean responses averaged over 40–60 and 100–120 min relative to baseline values and presented as a delta (i.e., average over 40–60 and 100–120 min post-drink period, respectively, minus the average over the 30 min baseline period). Drinks: water (W) 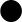, 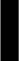; water + sugar (S) 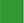, 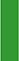; water + vodka (V) 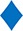, 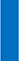; water + vodka + sugar (V+S) 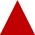, 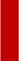. ^*^p < 0.05 significant differences over time from baseline values; ^$^*p* < 0.05 significant differences between responses to the drinks.

Figure 3 shows the changes over time for MBP, CO, TPR, and HR. Ingestion of the different drinks resulted in significant interaction effects (time × drink) for these parameters (*p* < 0.05). All drinks immediately raised MBP and TPR over baseline values. MBP was then above baseline values during the 120 min post drink with W and S. With V and V+S, MBP was not statistically different from baseline values from 20 to 40 min to the end of the test but was lower than after drinking W. TPR progressively decreased from 0–5 to 40–60 min with the greatest decrease observed after drinking V+S (−1.8 ± 0.2 mmHg.L^−1^.min). In contrast to W and V, drinking S and V+S raised CO over baseline values during almost all the 120 min post drink with a peak at 40–60 min (0.31 ± 0.07 and 0.39 ± 0.08 L.min^−1^, respectively). Over the first 60 min post drink, mean BrAC was correlated to mean CO (*R*2 = 0.22, *p* < 0.05). HR initially dropped below baseline levels (lower drop with V and V+S) and gradually increased during the first 60 min post-drink ingestion and then were stable until the end of the test. With W, HR stayed below baseline values over time. We observe the greatest increase in HR at both 40–60 and 100–120 min after V+S ingestion (6 ± 1 and 8 ± 5 beats.min^−1^, respectively). In addition, SV was increased immediately only after drinking W and S, with the greatest increase with S (W: 2.5 ± 0.7 mL.m^−2^; S: 4.6 ± 0.8 mL.m^−2^, *p* < 0.05). Then, SV stayed above baseline values over test with W while it gradually decreased to baseline values with S. SV was not altered after drinking V. After drinking V+S, SV slowly increased, peaking at 20–40 min (1.30 ± 0.5 mL.m^−2^) and progressively decreased to below baseline values at 100–120 min (−1.05 ± 0.6 mL.m^−2^).

**Figure 4:**
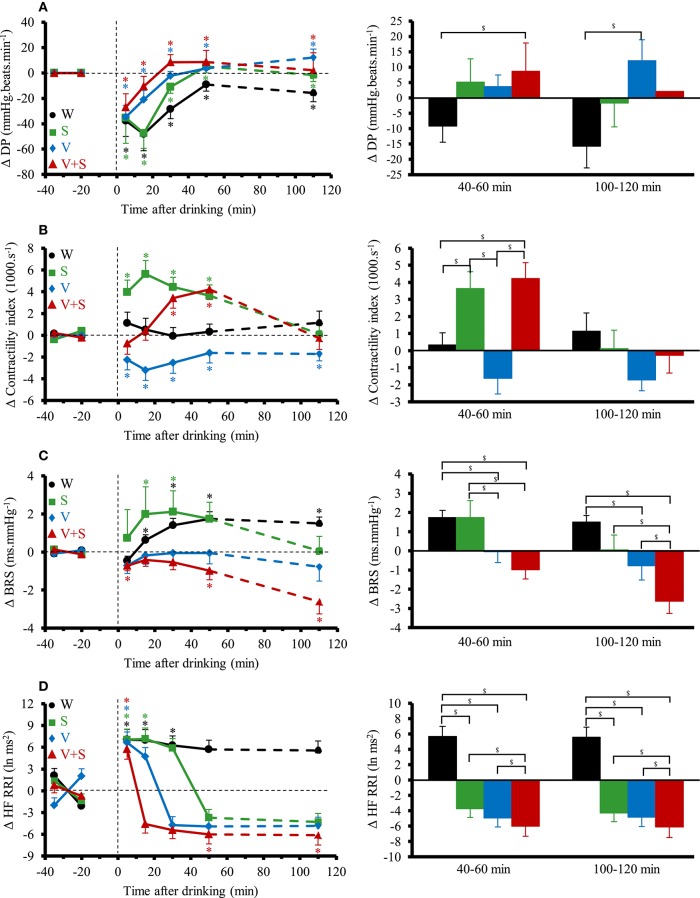
**(A–D)** Left panel: time course of the changes in double product **(A)**, contractility index **(B)**, baroreflex sensitivity (BRS) **(C)**, and high frequency power components of RR intervals (HF_RRI) **(D)**. Right panel: mean responses averaged over 40–60 and 100–120 min relative to baseline values and presented as a delta (i.e., average over 40–60 and 100–120 min post-drink period, respectively, minus the average over the 30 min baseline period). Drinks: water (W) 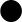, 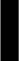; water + sugar (S) 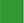, 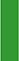; water + vodka (V) 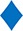, 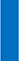; water + vodka + sugar (V+S) 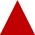, 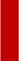. ^*^p < 0.05 significant differences over time from baseline values; ^$^*p* < 0.05 significant differences between responses to the drinks.

Figure 4 shows the changes over time for DP, contractility index, BRS and HF_RRI. DP immediately decreased with the four drinks. Averaged over the first 60 min post-drink, we observed differences in DP between drinks (W: −30 ± 7 mmHg.beats.min^−1^, S: −23 ± 9 mmHg.beats.min^−1^, V: −14 ± 17 mmHg.beats.min^−1^, −5 ± 28 mmHg.beats.min^−1^; W vs. V+S *p* < 0.05). We observed no differences after drinking V and V+S on DP. Immediately after drinking, contractility index was decreased with V and increased with S. Contractility stayed below baseline values during the 120 min post-drink ingestion with V whereas it was unaltered with W. After drinking V+S, contractility index progressively increased until 40–60 min and then decreased to baseline values at 100–120 min post-drink. BRS was increased after drinking W and S, unaltered with V and progressively decreased from 10–20 to 100–120 min with V+S (100–120 min: −2.6 ± 0.6 ms.mmHg^−1^). Immediately after W ingestion, HF_RRI was increased compared to baseline values (7.07 ± 1.4 ln ms2, *p* < 0.05) and was stable until the end of the test. The time course for HF_RRI after drinking S, V, and V+S showed an initial rise (S: 7.5 ± 1.4 ln ms2; V: 6.6 ± 1.5; V+S: 6.0 ± 1.4 ln ms2, *p* < 0.05) with a subsequent decrease under baseline values with V+S showing the largest drop compared to V and S at 40–60 and 100–120 min.

### Continuous cutaneous responses in sitting position

**Figure 5:**
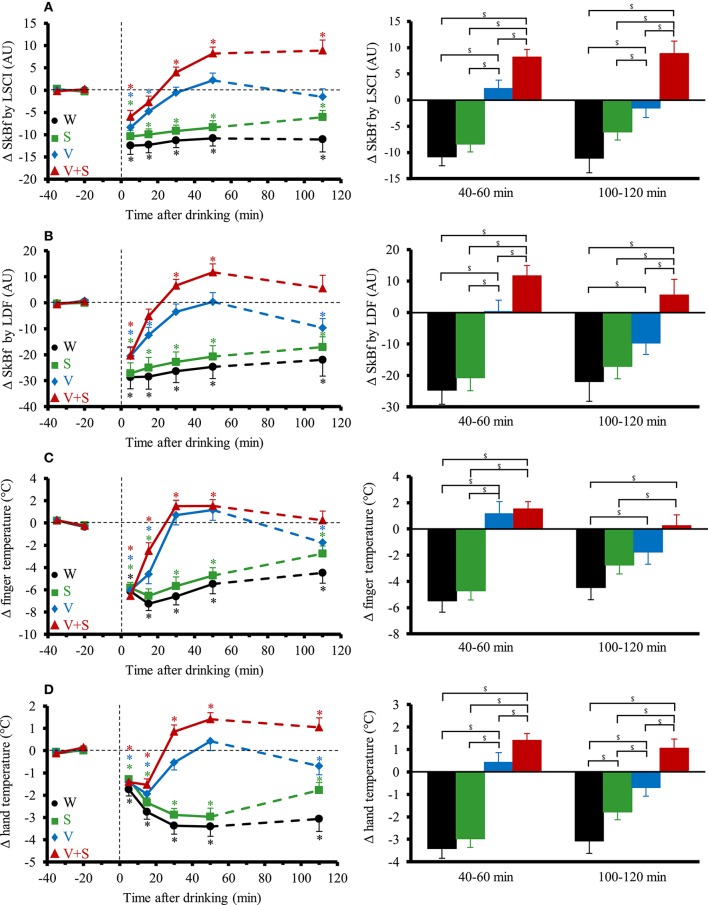
**(A–D)** Left panel: time course of the changes in skin blood flow (SkBf) by laser speckle contrast imaging (LSCI) **(A)**, by laser Doppler flowmetry (LDF) **(B)**, in finger temperature **(C)**, and hand temperature **(D)**. Right panel: mean responses averaged over 40–60 and 100–120 min relative to baseline values and presented as a delta (i.e., average over 40–60 and 100–120 min post-drink period, respectively, minus the average over the 30-min baseline period). Drinks: water (W) 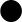, 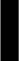; water + sugar (S) 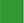, 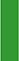; water + vodka (V) 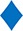, 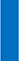; water + vodka + sugar (V+S) 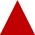, 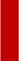. ^*^p < 0.05 significant differences over time from baseline values; ^$^*p* < 0.05 significant differences between responses to the drinks.

The time course and changes for SkBf and skin temperatures are shown in Figure 5. A significant interaction effect (time x drink) was found for all the parameters. SkBf and temperatures immediately dropped after drink ingestion with the four conditions. SkBf and temperatures stayed below baseline values over the 120 min post drink with the W and S. We observed a gradual increase in SkBf and temperatures from 0–5 to 40–60 min with V and V+S with a greater increase for V+S. SkBf and temperatures were still above baseline values 100–120 min post drink with V+S.

### Cardiovascular responses to active standing

**Figure 6:**
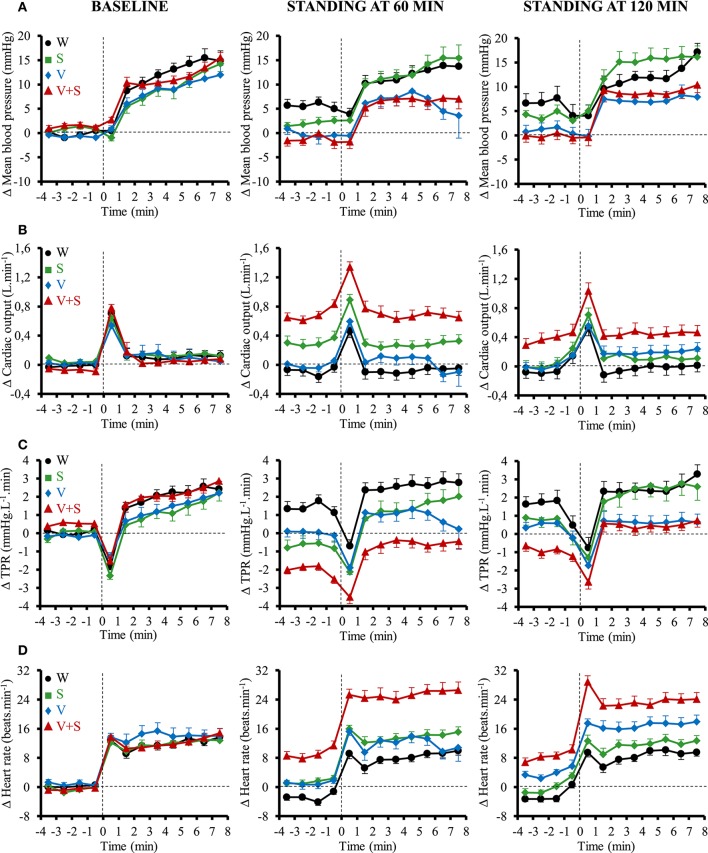
Time course of the changes in mean blood pressure **(A)**, cardiac output (CO) **(B)**, total peripheral resistance (TPR) **(C)**, and heart rate **(D)** 4 min before standing and during the first 8 min of active standing during baseline and after 60 and 120 min post-ingestion. Data are relative to baseline values prior to drink ingestion. Drinks: water (W) 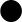, 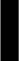; water + sugar (S) 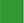, 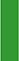; water + vodka (V) 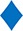, 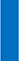; water + vodka + sugar (V+S) 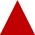, 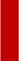.

The cardiovascular responses to orthostatic tests for MBP, CO, TPR, and HR are presented in Figure 6. Compared to the 4 min preceding the orthostatic test, the transient changes (i.e., first minute of active standing) for all these parameters were similar between all drinks. In the following 7 min, the changes in MBP, CO and TPR were also similar for all those conditions. However, there was a greater change in HR after drinking V+S during the next 7 min of the test at 60 min (W: 10 ± 1, S: 12 ± 1, V: 11 ± 1, V+S: 16 ± 1 beats.min^−1^, *p* < 0.05) and 120 min (W: 11 ± 1, S: 11 ± 1, V: 13 ± 1, V+S: 15 ± 1 beats.min^−1^, *p* < 0.05).

However, to put forward the actual cardiovascular load of the combination drink and active standing we also expressed the absolute values. During the first minute of active standing at 60 min post drink ingestion, we observed a greater CO (W: 5.1 ± 0.1, S: 5.4 ± 0.1, V: 5.0 ± 0.1, V+S: 5.9 ± 0.1 L.min^−1^, *p* < 0.05) and HR (W: 68 ± 2, S: 72 ± 2, V: 70 ± 2, V+S: 81 ± 2 beats.min^−1^, *p* < 0.05) and a lower TPR (W: 16.9 ± 0.6, S: 15.6 ± 0.5, V: 16.5 ± 0.6, V+S: 13.0 ± 0.3 mmHg.L^−1^.min, *p* < 0.05) after drinking V+S. During the following 7 min, we also found a higher CO (W: 5.1 ± 0.1, S: 5.3 ± 0.1, V: 5.1 ± 0.1, V+S: 5.8 ± 0.1 L.min^−1^, *p* < 0.05) and HR (W: 77 ± 2, S: 83 ± 2, V: 81 ± 2, V+S: 94 ± 2 beats.min^−1^, *p* < 0.05), and a lower TPR (W: 17.9 ± 0.5, S: 17.3 ± 0.5, V: 17.3 ± 0.4, V+S: 14.5 ± 0.4 mmHg.L^−1^.min, *p* < 0.05) after the ingestion of V+S.

### Gender differences

As stated above, we did not find gender differences in BrAC. We observed no gender effect of alcohol on the cardiovascular and cutaneous responses both in sitting position and during orthostatic tests except a slightly higher CO after V+S and S in women between 40 and 60 min post drink ingestion.

## Discussion

The aim of the present work was to evaluate the interaction of alcohol consumption with sugary drinks in healthy young male and female subjects on the cardiovascular system and on the cutaneous response. Our findings reveal that compared to alcohol alone, the interaction alcohol and sugar induced (1) a lower BrAC; (2) hemodynamic changes in sitting position with a lower BP and TPR, a higher CO and HR, a greater increase in SkBf and hand temperature, and a decrease in BRS; (3) a greater tachycardic response during active standing and (4) similar results between men and women both in sitting position and during prolonged active standing.

### Alcohol metabolism and BrAC

When alcohol is consumed, it passes from the gastrointestinal tractus (i.e., from the stomach and intestines) into the blood, a process referred as absorption. Some of the alcohol which is ingested orally does not enter the systemic circulation but is oxidized mainly in the stomach (primary pathway involving gastric alcohol dehydrogenase enzymes) (Frezza et al., 1990), decreasing the bioavailability of alcohol (first pass metabolism) and thus BrAC. First pass metabolism of alcohol is modulated by many factors such as activity of gastric alcohol dehydrogenase enzymes (Frezza et al., 1990) and the speed of gastric emptying (Oneta et al., 1998). Delayed gastric emptying increases the time of exposure of alcohol to gastric alcohol dehydrogenase enzymes, increasing first pass metabolism of alcohol, leading to a lesser absorption and thus a lower BrAC. Previous studies already exposed higher BrAC in humans after drinking alcohol mixed with artificially sweetened (i.e., diet) compared to sugar-sweetened (i.e., regular) soft drinks (Wu et al., 2006; Marczinski and Stamates, 2013; Stamates et al., 2015). Sugars in sweet drink reduces the gastric emptying rate (Kalant, 1971) explaining the lower BrAC after drinking V+S compared to V observed in the present study.

### Hemodynamic changes with sugar and alcohol ingestion

We found an immediate depression of HR immediately after ingestion of the 4 drinks followed by a gradual increase until 40–60 min. This initial drop can be explained by alterations in autonomic function. Indeed, we already reported that ingestion of 500 mL of water leads to an activation of vagal tone (Brown et al., 2005; Girona et al., 2014), which depends on drink temperature, cooler drinks leading to a higher vagal tone (Girona et al., 2014). In the present study, the higher HF_RRI observed immediately after ingestion of the 4 drinks at around 10°C supports this hypothesis. Interestingly, the greatest rise in HR concomitant with the larger decrease in HF_RRI was observed after V+S despite a lower BrAC. Thus it seems that tachycardia was due to the combining effects of sugar and alcohol rather than alcohol alone. The increased HR could be explained by the withdrawal of vagal tone (Koskinen et al., 1994; Spaak et al., 2010) with alcohol and by the stimulation of sympathetic nervous system induced both by sucrose (through insulin secretion, Brown et al., 2008) and alcohol (Iwase et al., 1995; Randin et al., 1995; van de Borne et al., 1997; Hering et al., 2011).

After a stable period, the small rise in SV at 20–40 min after V+S was only due to sucrose since SV was unaltered after V whereas it increased during the 60 min following ingestion of S. It seems that the increased cardiac contractility after ingestion of sucrose with insulin secretion, Muniyappa et al. (2007) was counterbalanced by the well-described negative effects of alcohol on this parameter (Kelly et al., 1996). This hypothesis was strengthened by the increased contractility observed only from 20 to 40 min. In our study, CO was similar between V and W whereas the rise observed with S was even more pronounced with V+S suggesting that alcohol emphasized the effects of sucrose on CO. The correlation we found between mean BrAC over the first 60 min post-drink and CO (*R*2 = 0.22, *p* < 0.05) strengthens this hypothesis. It has been well-described that sugar increases CO (Brown et al., 2008) through an insulin-mediated elevation in cardiac contractility and HR, and a decrease in peripheral resistance (Muniyappa et al., 2007). Alcohol intake increases gastroduodenal permeability, thereby increases sucrose absorption, (Keshavarzian et al., 1994), acutely augments glucose-stimulated insulin secretion (Adner and Nygren, 1992) and increases insulin sensitivity (Avogaro et al., 2004). All these effects may contribute to the greater CO with the combination of alcohol and sugar.

Compared to alcohol alone, the greater decrease in MBP after the combination of alcohol and sugar was accompanied by a higher increase in CO and a larger drop in TPR. Concomitantly, we observed a greater rise in SkBf and hand and finger temperature. Here, we show that the combination of alcohol and sugar (i.e., V+S) seems to exacerbate the peripheral skin vasodilation already observed after alcohol (Bau et al., 2005) alone. How exactly alcohol alone and its interaction with sugar (through insulin secretion) causes peripheral vasodilation remains uncertain. Both alcohol (Steiner et al., 2015) and sugar (Blaak and Saris, 1996) induce insulin secretion. Vasodilator effects of insulin include dilation of terminal arterioles (decrease in SkBf) and relaxation of larger resistance vessels, leading thus to a decrease in TPR (Muniyappa et al., 2007).

Peripheral dilation induced both by insulin (Taddei et al., 1995; Muniyappa et al., 2007) and alcohol alone (Takahashi et al., 2008; Rocha et al., 2012) are mediated by neurohormonal substances including nitric oxide and the sympathetic nervous system. Further studies are needed to clarify the effect of the combination of their ingestion.

### Combination of alcohol and sugar during active standing

Previous studies already reported impairment of the vasoconstrictor response to orthostatic head up tilt test after alcohol ingestion (Narkiewicz et al., 2000; Carter et al., 2011). In contrast to those authors, we used active standing and a more moderate dose of alcohol (0.4 g.kg^−1^ of body weight instead of 0.8–1.0 g.kg^−1^ of body weight). We observed that, compared to the 4 min preceding the active standing, alcohol alone (i.e., V) did not induce major changes in BP, CO, TPR, and HR compared to non-alcoholic drinks. However, the combination of sugar and alcohol (i.e., V+S) induced a greater tachycardia during the orthostatic tests. For orthostatic test, we did not use head up tilt test but prolonged active standing because it mimics the real-life situations.

Although the combination of sugar and moderate dose of alcohol did not blunt the vasoconstrictor response to active standing in our study, it may encroach upon the hemodynamic reserve to a further cardiovascular challenge. Indeed, because of the altered baseline values before active standing after drinking V+S, when we expressed the orthostatic responses as absolute values, we observed a greater vasodilation, tachycardia and CO and a lower MBP. In this context, subjects could be more sensitive to an additional hypotensive and vasodilatory stimulus such as emotional stress (Sharpey-Schafer et al., 1958), heat exposure (Wilson et al., 2006) or after exercise (Halliwill et al., 2013). It would be very interesting to analyze the effects of the combination of alcohol and sugar in a more complex situation for the subjects to emphasize the hemodynamic challenge in order to mimic the real-life situation of young people for example when combining alcohol consumption with physical exertion and environmental stress (e.g., during a “party” through dancing in locations with high temperatures).

### Gender differences

Gender-differences exist for alcohol metabolism, mainly due to a smaller gastric metabolism in females (because of a significantly lesser activity of gastric alcohol dehydrogenase enzymes) (Frezza et al., 1990; Baraona et al., 2001). Discrepancies exist between studies reporting either higher (Frezza et al., 1990) or similar (Lucey et al., 1999) blood alcohol level between men and women when doses are adjusted for bodyweight. In the present study, we did not find any sex-differences on BrAC at each time point or on the average over the test. Baraona et al. (2001) showed that women had less first pass metabolism than men when given alcohol in high (i.e., 40%) but not in low (5%) concentration. The concentration-dependency of these effects may explain earlier discrepancies. Thus, the similar BrAC observed between men and women in our study is most likely explained by the small concentration of alcohol in the drink ingested (i.e., 10.2 ± 0.4%). In this context, all groups were pooled because of the minor differences in cardiovascular and cutaneous responses between men and women that may be explained by their similar BrAC. For a similar BrAC in men and women, there was no gender differences on the cardiovascular response to alcohol ingestion both in sitting position and during active standing except for CO. Since we found no differences in BrAC between genders, the slightly higher CO observed in women with V+S can be explained by their higher insulin secretion observed after glucose ingestion (Basu et al., 2006) and the higher quantity of sugar received on a kg basis. Indeed, with sugar alone, CO was also slightly higher in women.

### Limitations

There are some limitations in the present study. First, in our study, we used moderate doses of alcohol (i.e., 0.4 g.kg^−1^ of body weight). We can speculate that higher doses of alcohol could emphasize the impairment of the hemodynamic response to active standing and induce orthostatic hypotension. Second, we recruited only healthy individuals allowing a more homogeneous sample, but we did not test the potential impact in older people or in people with pre-existing heart diseases. Finally, alcohol concentration was measured in expired breath and not with blood samples to avoid stress for subjects. However, many studies highlighted a high correlation between blood alcohol concentration and BrAC (Lindberg et al., 2007; Jaffe et al., 2013).

## Conclusion

In our study, we show that, despite lower BrAC after V+S compared to V, ingestion of the combination of alcohol and sugar induces hypotension in sitting position because of acute vasodilation and an impairment of the hemodynamic reserve during active standing compromising the orthostatic tolerance. The underlying mechanisms are not fully elucidated but could be related to the interaction between insulin and alcohol. Even if subjects did not feel any signs of syncope, these results could be of clinical importance with higher doses of alcohol or if combined to other hypotensive challenges. We also found that for a similar BrAC in men and women, there was no gender differences on the cardiovascular response to alcohol ingestion both in sitting position and during prolonged active standing.

## Author contributions

CM: Conceived and designed research, contributed to acquisition, analysis and interpretation of data, drafting of the manuscript, and critical revision of the manuscript for important intellectual content. NC: Conceived and designed research, contributed to acquisition, analysis and interpretation of data and critical revision of the manuscript for important intellectual content. J-PM: Conceived and designed research, contributed to analysis and interpretation of data, drafting of the manuscript, and critical revision of the manuscript for important intellectual content.

### Conflict of interest statement

The authors declare that the research was conducted in the absence of any commercial or financial relationships that could be construed as a potential conflict of interest.

## Abbreviations

BP: blood pressure
BrAC: breath alcohol concentration
BRS: baroreflex sensitivity
CO: cardiac output
DBP: diastolic blood pressure
DP: double product
HR: heart rate
LDF: laser Doppler flowmetry
MBP: mean blood pressure
SBP: systolic blood pressure
SkBf: skin blood flow
SV: stroke volume
TPR: total peripheral resistance.
